# Domestic violence on children: development and validation of an instrument to evaluate knowledge of health professionals**^1^**

**DOI:** 10.1590/1518-8345.0805.2772

**Published:** 2016-08-18

**Authors:** Lanuza Borges Oliveira, Fernanda Amaral Soares, Marise Fagundes Silveira, Lucinéia de Pinho, Antônio Prates Caldeira, Maísa Tavares de Souza Leite

**Affiliations:** 2 MSc, Professor, Faculdades Integradas Pitágoras de Montes Claros, Montes Claros, MG, Brazil.; 3 Undergraduate student in Medicine, Faculdades Integradas Pitágoras de Montes Claros, Montes Claros, MG, Brazil; 4 PhD, Professor, Universidade Estadual de Montes Claros, Montes Claros, MG, Brazil.

**Keywords:** Validation Studies, Child Health, Health Personnel, Domestic Violence, Child Abuse

## Abstract

**Objective:**

to develop and validate an instrument to evaluate the knowledge of health professionals about domestic violence on children.

**Method:**

this was a study conducted with 194 physicians, nurses and dentists. A literature review was performed for preparation of the items and identification of the dimensions. Apparent and content validation was performed using analysis of three experts and 27 professors of the pediatric health discipline. For construct validation, Cronbach's alpha was used, and the Kappa test was applied to verify reproducibility. The criterion validation was conducted using the Student's t-test.

**Results:**

the final instrument included 56 items; the Cronbach alpha was 0.734, the Kappa test showed a correlation greater than 0.6 for most items, and the Student t-test showed a statistically significant value to the level of 5% for the two selected variables: years of education and using the Family Health Strategy.

**Conclusion:**

the instrument is valid and can be used as a promising tool to develop or direct actions in public health and evaluate knowledge about domestic violence on children.

## Introduction

The concept of violence includes neglect, psychological violence and sexual abuse, in addition to physical abuse^1^. Domestic violence is a multifactorial phenomenon, consisting of many variables that affect all societal levels and requires intervention from a multidisciplinary team to enable comprehensive care for the victim. It is also a particularly painful reality when it is perpetrated on children. In this case, domestic violence is one of life's events that can definitely change the child's behavior over the long term. The consequences for children can be immediate, medium and long term, and the feelings generated by pain resulting from such acts are most often repressed, forgotten, denied, but never disappear. Psychological trauma can develop, negatively affecting the personality over the entire life, or trigger hostile attitudes, distrust and fear^2^ ^-^ ^3^. 

National and international data on domestic violence on children show the relevance of the problem. Beginning in 2006, Brazil implemented the Violence and Accident Surveillance System - Vigilância de Violências e Acidentes (VIVA)^4^. One study conducted with health professionals reported that 69.5% of them cared for cases of infant-juvenile violence: 60.0% asked for advice from another professional before notification, 54.0% talked with family members, and 42.9% reported the incident in the VIVA system^5^. 

The diagnosis of violence in childhood is difficult, as children tend to hide the real cause of the injuries either due to fear or love, since the aggressors are usually the parents or guardians. Thus, professionals who deal with this group, especially health care professionals, must be attentive to details that can confirm the diagnosis^6^. Notification is not a personal act, but a legal one. Typically, health care professionals are the first to detect the violent situation and considering this fact, must immediately notify when it is observed. When the health professional does not recognize himself in this role, it becomes an impeding factor for referral and timely treatment of victims of domestic violence^1^ ^-^ ^7^. 

The expansion of primary care services that recently occurred in Brazil, by increasing the number of Family Health Strategy (FHS) teams, is an opportunity for greater surveillance of domestic violence. In theory, these teams should be able to deal with situations of violence, but little is known about the knowledge on this subject, or the ability of team members to approach the problem. Expanding the capacity to detect violent situations is necessary to allow the construction of social support networks and confrontation of these situations^8^.

Given the complexity of this problem, its approach cannot be competence a single area of or professional category. This complexity involves a multidisciplinary view and intersectoral action, as part of collective actions. Within the FHS framework, information can be produced and exchanged with those of other sectors. Thus, professionals must employ means such as social networking, reflecting on the vulnerable conditions of life, aiming to ensure rights, in the same way as developing potential actions against violence upon children^9^. 

The knowledge of health professionals on this issue must be evaluated. However, effective tools to assess this knowledge are not available. Although tools for evaluation of the victims of psychological, physical and sexual violence are available, instruments aiming to evaluate health professionals were not found^10^ ^-^ ^11^. Further studies are necessary to verify the knowledge of professionals that guide public health policies, and to combat domestic violence in children. This study aimed to develop and validate an instrument that evaluates the knowledge of health professionals about domestic violence in children.

## Method

**Figure 1:**
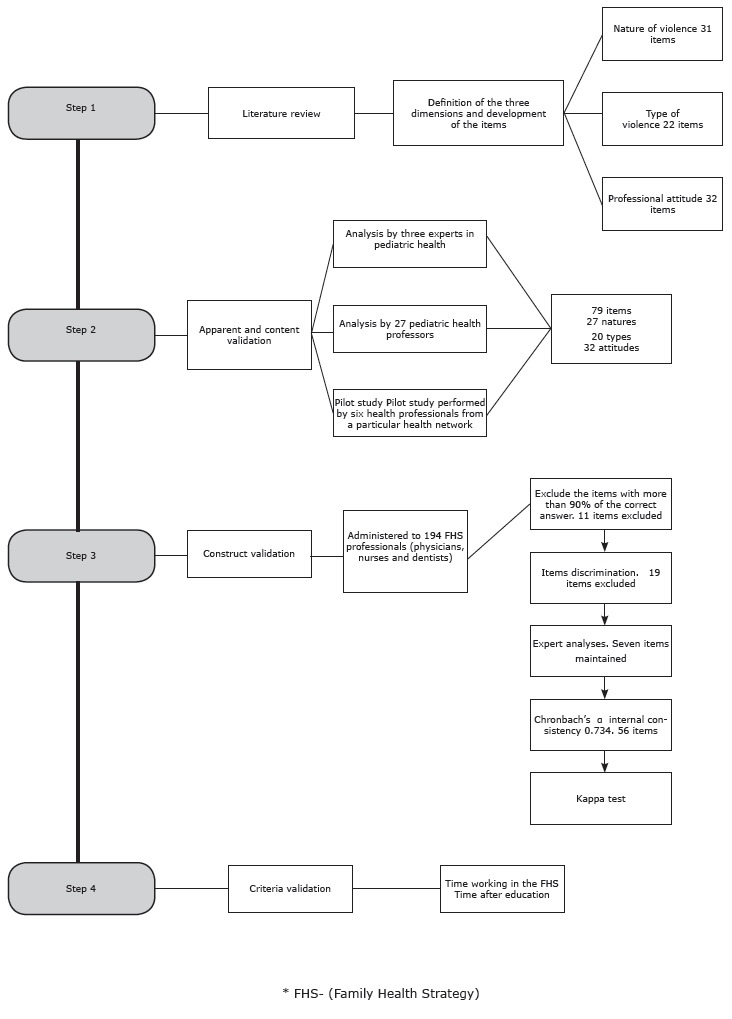
Flowchart development and validation steps of the instrument "Knowledge about domestic violence in children in the practice of healthcare professionals"

The study consisted of the development and validation of an instrument for evaluating the knowledge about domestic violence in children. The process was conducted in four steps: 1) identification of the dimensions related to domestic violence in children; 2) apparent and content validation; 3) construct validation; and 4) criteria validation, as shown in Figure 1.

### Step 1- Identification of the dimensions related to domestic violence on children

The identification of the instrument's items related to "Knowledge about domestic violence in children in the practice of healthcare professionals" was based on education materials/articles and on national and state guidelines on the theme^12^ ^-^ ^13^. An integrative literature review was conducted using the descriptors *child health, domestic violence, health personnel and family health*. The search was conducted between March and May of 2013, in the Virtual Health Library; in the databases: Literatura Latino-Americana em Ciências da Saúde (LILACS), *Medical Literature Analysis and Retrieval System Online* (MEDLINE) and *Scientific Electronic Library Online* (SciELO). The inclusion criteria for this study included articles in Spanish, English or Portuguese language, which analyzed the approach of healthcare professionals, regarding domestic violence on children, published from 2009 to 2013.

**Figure 2:**
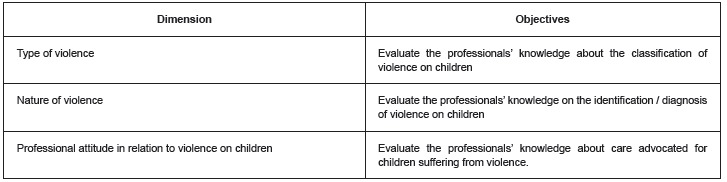
Objectives of the instrument dimensions, "Knowledge about domestic violence on children in the practice of healthcare professionals"

The dimensions established as important and relevant were identified, and the operational objectives were defined. The synthesis of the main contents resulted in 85 items, grouped in three dimensions: 31 items for the *nature of violence* dimension, 22 items for *type* of *violence,* and 32 items for *professional conduct in relation to violence on children*. The three dimensions considered in the study and their objectives are presented in Figure 2.

The main themes within each dimension were identified and transformed into short and objective statements, which constituted the items. Part of these was maintained as true statements, similar to the reference text; and the other part was transformed into false statements. After each statement, response options were organized in a Likert type scale of three levels: *agree, disagree,* and*, I don't know.*

### Step 2- Apparent and content validation

The instrument was subjected to content and semantic structure analysis by three experts in the area: a physician with a doctorate in pediatrics and two nurses with a doctorate, experts in pediatric health, who evaluated the presence or absence of the comprehensiveness, objectivity and relevance criteria. The instrument was also administered to 27 professors of undergraduate and graduate programs in the health area that taught disciplines related to child health. After the analysis of experts and professors, the instrument was redesigned, according to directions and suggestions, and six items were excluded. Next, a pilot study was performed with six health professionals from a private health system the municipality, in order to verify the adequacy and clarity regarding the interpretation of the instrument.

### Step 3- Construct validation

The preliminary version of the instrument, "Knowledge about domestic violence on children in the practice of health professionals" with 79 items, was administered to 194 professionals (physicians, nurses and dentists) registered at the FHS of Montes Claros, MG.

Those items correctly answered by more than 90% (very easy) and less than 10% (considered too difficult level) of the FHS professionals were excluded^14^. The correlation of each item with the mean score of the full questionnaire was performed, using the minimum cutoff point of 0.2 for the correlation coefficient between the total mean score and each item of the instrument, excluding those items with lower values^15^. After expert and researcher analysis, seven items considered important with respect to content were maintained. As the internal coherence decreased when dimensions classified the instrument, it was considered as a single scale; thus, the Chronbach α value was calculated for the full instrument with 56 items, reflecting a satisfactory level of internal consistency^16^. After this analysis, the reproducibility of the questionnaire was evaluated by administering the redesigned version to 30 professionals among the 194, with an interval of two weeks. The *Kappa* test was applied to verify the correlation between the mean scores of the test-retest of the instrument items, and for the Kappa agreement test, the following parameters were used: small agreement <0.40; moderate agreement: 0.41-0.60; good agreement: 0.61-0.80; excellent agreement > 0.80^17^.

### Step 4- Criteria validation

Initially, the scores were calculated using the sum of the values assigned in the Likert scale of the items included in the instrument by assigning the following values for statistical purposes: zero to answer "I don't know", +1 for a correct answer and -1 for a wrong answer. Using the formula of total amplitude, which is the difference between the highest and the lowest value from a data set (W = Xn-X 1), the transformation of the scores on a scale of 0 to 100 was performed. In this case, the highest value is represented by X_n_, and X_1_ for the smallest value. For this instrument, the total amplitude would be equal to 112, because W=56-(-56). Thus, with the use of the formula Y=(X+56) x 100/112, the transformation of each score in centesimal scale was obtained, where X is the final score of each questionnaire, which can range from -56 to + 56; and Y is the final value of the scale from 0 to 100^18^.

For classification of the level of knowledge, the scale of 0 to 100 was used, and the score of 70 or more points would be considered satisfactory, and scores of less than 70 points would be considered unsatisfactory.

The comparison of scores of items was performed among the subgroups that hypothetically should present different levels of knowledge, using the *student t*-test, with a significance level of 5%. These groups were defined based on " time since graduation" and "time working in the FHS". These variables were dichotomized considering the overall mean time at the FHS and time working, in order to verify the degree of effectiveness of the instrument in predicting specific performance of the subject^19^. 

The data was analyzed using the SPSS IBM statistical software program, version 19. The study was conducted, conforming to the ethical principles for research involving human beings. The participation was voluntary, the informed consent form was signed, and the Research Ethics Committee of the Universidade Estadual de Montes Claros, protocol No. 401,241/2014, approved the design of the study.

## Results

Among the professionals who participated in the validation process, a predominance of women (74.7%) was verified; nurses (42.8%), with less than five years since graduation (55.6%). The title that was most often identified was that of family health resident (37.6%). Most of them had one to five years working in the FHS (44.3%), and almost half (45.4%) had attended cases of domestic violence on children.

While developing the instrument, the summary of the issues identified in the literature resulted in 85 items. After analysis of the experts and professors, six items were excluded, and the remaining 79 were arranged at random, according to the numeric Likert scale of three levels, with the following options: agree, disagree, I don't know. For construct validation, after analyses by 194 professionals, 11 more items were excluded that were considered too easy or too hard, that is, as the agreement and errors by more than 90% of the professionals. Among that, 56 items remained, whose Cronbach's α analysis was 0.734, reflecting a satisfactory level of internal consistency.

Table 1 presents the result of the Kappa statistic in the reproducibility of the instrument. More than 80% of the items showed a moderate to very good agreement^12^.

**Table 1:** Kappa statistic for the reproducibility of the instrument. Montes Claros, MG, Brazil, 2014

Kappa	Items
<0.40	2,7,14,21,22,23,27,42,51,52,55
0.41 - 0.60	9,17,20,24,26,29,30,32,34,35,36,38,39,41,45,46,47,48,49,53,54
0.61 - 0.80	1,3,4,5,6,8,10,11,12,13,15,16,18,25,28,31,33,43,44,50,56
> 0.80	19,40

The results of criteria validation, based on the time working in FHS and time since completing education are shown in Table 2. Both the criteria used showed a statistically significant association.

**Table 2:** Mean score of time in the FHS and time after educational degree of professionals. Montes Claros, MG, Brazil, 2014

Variables		Mean (SD^?^)	p-valor
Time working in FHS			
	Up to 3 years	70.40 (8.75)	0.039
	>3 years	73.02 (8.76)	
Time after educational degree			
	Up to 5 years	70.44 (8.79)	0.037
	>5 years	73.09 (8.70)	

*SD: standard deviation

## Discussion

The development of a reliable and valid instrument occurred according to elements considered important. The instrument evaluates the knowledge about domestic violence on children in the practice of health professionals, considered important for implementation of actions that improve the care of the child victim of domestic violence, a reality in several family nuclei.

The first bibliographic survey showed the absence of an instrument that evaluates the knowledge about domestic violence of the health professionals. Schraiber et al., conducted a study with the objective of validating the instrument, *World Health Organization Violence against Women* (WHO VAW) on psychological, physical and sexual violence by intimate partners against women^10^. Authors translated, adapted and validated the content of the *Childhood Trauma Questionnaire*, which is administered to adolescents and adults, with the aim to investigate the history of abuse and neglect during childhood^11^. 

The process of preparation followed the recommended methodological and statistical aspects^20^ ^-^ ^22^, with satisfactory internal consistency and reliability in the final version. According the recommended aspects in the *development of items,* as performed in the study of Paschoal and Tamayo, the items were prepared based on the literature^23^, to *validate the content*. 

Semantic analysis was performed with a more sophisticated sample (higher skill) of the studied population, ensuring that the items in the instrument were intelligible to the lower stratum. Thus, the instrument was referred for an analysis of experts on the subject, suggesting addition or modification of items. This same process was conducted in the study of Stelkoo-Pereira and colleagues, whose goal was the validity and internal consistency of the *School Violence Prevalence Investigation Questionnaire - student version,* a situation similar to that which was found in the study of Hermida and Araújo that aimed for the development and validation of the nursing instrument^24^ ^-^ ^25^. 

In *construct validation*, the dimensions of the items of the instrument of this study, must be simultaneously evaluated, as the internal coherence decreases when they are individually analyzed, and the value of *Cronbach's α* indicates a positive association when a larger number of issues is associated. Similar results were obtained in the survey conducted by Pinho and collaborators^26^. The *criteria validity* showed significance when the time since completion of education and the time working in the FHS, according to the knowledge, were analyzed, as the performance of the research subject and actual behavior are related.

In the studied context, the instrument developed has been shown to be important, since it can be used for verification of the knowledge of working professionals, and can be used by professors in undergraduate health courses, whose theme is present, as a learning tool.

Investment in education, providing the professional with tools for early identification of cases of violence on children is necessary^27^. The importance of the health care professional and members of the FHS teams being attentive for the detection of children who are victims of violent acts, showing discernment and responsibility for notification regarding cases, even if they are only suspicions, is also stressed^28^. 

## Conclusion

The final version of the questionnaire showed a satisfactory internal consistency and good reliability and reproducibility, as shown in the Cronbach's alpha, Kappa test and student's t-test statistics.

The instrument on the evaluation of knowledge about domestic violence on children, in the practice of health professionals, was valid and still awakens the interest of the health care professional on the subject, which can be regarded as positive and encourages an approximation with the subject. Furthermore the instrument is simple, objective, relatively short and easy to understand and can be considered as a promising tool to develop or direct actions in public health and public policy of intervention with respect to domestic violence on children, and can be used by teachers as a learning tool. The instrument is available for use in further studies. 
